# Unskilled and Don't Want to Be Aware of It: The Effect of Self-Relevance on the Unskilled and Unaware Phenomenon

**DOI:** 10.1371/journal.pone.0130309

**Published:** 2015-06-12

**Authors:** Young-Hoon Kim, Chi-Yue Chiu, Jessica Bregant

**Affiliations:** 1 Department of Psychology, Yonsei University, Seoul, South Korea; 2 Department of Psychology, The Chinese University of Hong Kong, Hong Kong, China; 3 Department of Psychology, University of Chicago, Chicago, Illinois, United States of America; Northwestern University, UNITED STATES

## Abstract

Previous research found that poor performers tend to overestimate how well their performance compares to others’. This unskilled and unaware effect has been attributed to poor performers’ lack of metacognitive ability to realize their ineptitude. We contend that the unskilled are motivated to ignore (be unaware of) their poor performance so that they can feel better about themselves. We tested this idea in an experiment in which we manipulated the perceived self-relevancy of the task to men and women after they had completed a visual pun task and before they estimated their performance on the task. As predicted, the unskilled and unaware effect was attenuated when the task was perceived to have low self-relevance.

## Introduction

When people estimate their performance on a task relative to a group, those who perform the worst tend to overestimate their performance, while those who perform the best actually underestimate their performance [1–4]. For example, Kruger and Dunning [3] found that participants whose performance fell in the lower quartiles tended to overestimate their relative performance, whereas participants whose performance fell in the upper quartiles tended to underestimate their relative performance.

Kruger and Dunning [3] (see also [5]) offered a cognitive interpretation of these results, arguing that the poor performers simply lack the necessary metacognitive expertise to realize their ineptitude. They argued that “incompetence, like anosognosia, not only causes poor performance but also the inability to recognize that one’s performance is poor” (p. 1130). They further stated that “bottom-quartile participants were less successful than were top-quartile participants in the metacognitive tasks of discerning what one has answered correctly versus incorrectly (Study 4) and distinguishing superior from inferior performances on the part of one’s peers (Study 3)”. Thus, Kruger and Dunning referred to this judgment phenomenon as the *unskilled and unaware effect*.

**Fig 1:**
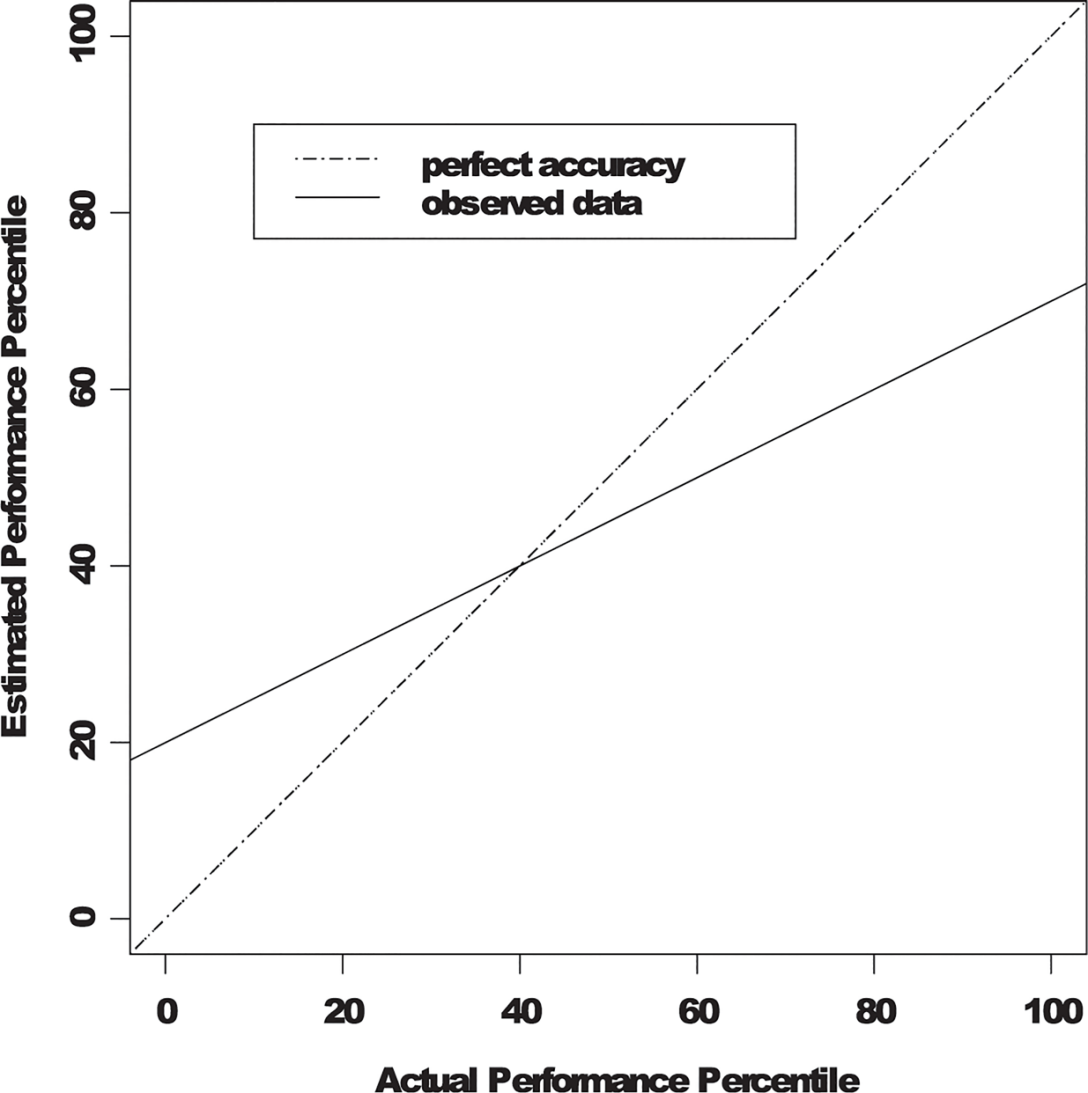
The unskilled and unaware phenomenon.

However, the metacognitive approach overlooks the potential role of motivation in driving this effect. There may also be a motivational component to the unskilled and aware effect itself. To alternatively represent these data, Krueger and Mueller [4] plotted participants’ estimated performance percentile ranks against their actual performance percentile ranks and fitted a regression line. Fig 1 depicts the typical pattern where individuals’ estimated performances relative to the group (in percentile scores) are plotted against their actual relative performances in the group (also in percentile scores) and a regression line relating actual performance to self-assessment of ability is fitted (see, e.g., Krueger & Mueller [4]). The unit line in Fig 1 represents perfect accuracy with no response bias (intercept of 0) and perfect estimation accuracy (slope of 1). In this hypothetical scenario, both high and low performing individuals estimate their performance accurately. The unskilled and unaware effect could be represented by solid line: it has a significant positive intercept (intercept > 0), indicating the presence of a positive response bias and a slope that is significantly higher than 0 but lower than 1, indicating some sensitivity to actual performance but imperfect accuracy when making performance estimation. These two statistical parameters, the intercept and the slope, jointly contribute to the overestimation of low performing individuals and the underestimation of high performing individuals. That is, the combination of a positive response bias (as reflected in a positive intercept) and a less-then-perfect estimation accuracy (as reflected in a slope higher than 0 but lower than 1) contributes to the appearance that low performing individuals overestimate their performance and high performing individuals underestimate theirs.

While the metacognitive explanation fits these data, the results (as depicted in Fig 1) are also consistent with an alternative explanation: self-enhancement motivation. Because individuals are motivated to self-enhance [6–8], they display a positive response bias (as reflected in the positive intercept in the regression line) and do not consider their actual performance fully when estimating performance (as reflected in non-unit regression slope). The intercept in the regression line has been proposed to represent response bias [4]. Under a motivational account, this response bias is more than a simple lack of metacognitive awareness; it reflects a self-enhancement motivation that elevates participants’ self-assessment. Under the same account, the slope in the regression line reflects the extent to which people base their self-assessment on their actual performance. When people consider and base ability judgments exclusively on the task performance, the slope would be nearly parallel to the unit line. In contrast, the slope would be nearly flat when people do not consider their actual task performance in estimating performance.

If unskilled and unaware effect has a motivational component, then the effect should be affected by manipulations that influence motivation. It is well-documented that the self-enhancement motive is stronger when the task is perceived as relevant to self-evaluation than when it is not (see [9]). Therefore, according to the self-enhancement account, when the task is perceived to have low (vs. high) self-relevance, the self-enhancement motive would be weakened. Under this circumstance, individuals may exhibit an attenuated (positive) response bias and base estimation of their performance on their actual performance more. By the same logic, when the task is perceived to have high self-relevance, the self-enhancement motive would be strengthened. Under this circumstance, individuals may display a stronger (positive) response bias and dissociate their estimation from their actual performance more. However, according to the metacognitive account of the unskilled and unaware effect, unskilled performers are inaccurate because they lack insight into their incompetence. Thus, whether or not they perceive the task to be self-relevant should not impact their estimation of their performance on the task. These contrasting hypotheses form the basis for the present study, the primary objective of which is to test this self-enhancement account of the unskilled and unaware effect.

## Predictions and Hypotheses

To examine this idea, we had participants perform a task and then estimate their performance relative to others’ in the group. Just before they made the estimation, they were led to believe either that the task would be equally relevant to men and women or that the task would be less relevant to men than women. We hypothesize that when the task was perceived to be equally relevant to men and women, female and male participants would exhibit the typical unskilled and unaware effect to a similar extent. However, when the task was perceived to be less relevant to men than to women, the unskilled and unaware effect would be attenuated among men, resulting in a weaker unskilled and unaware effect among men than women.

## Method

### Ethics Statement

The Study was approved by the Institutional Review Board for the Protection of Human Subjects at the University of Illinois at Urbana-Champaign. All participants voluntarily filled out an informed consent form agreeing to participate in the study.

### Participants

One hundred forty-three participants (86 females) were recruited from a public university in the United States. The age of participants ranged from 18 to 49 (*M* = 20.09, *SD* = 2.75). Participants received extra credit toward their class for their participation.

### Materials

A visual pun task (http://www.rwmarketing.com/news/vispun.html) was used in the current study. The task consists of 10 trials. On each trial, a symbol (e.g., “history history history”) that suggests a common phrase or cliché (e.g., “history repeating itself”) was presented, and the participants’ task was to identify the phrase or cliché. We chose this task because it appears to measure many different abilities (e.g., language ability, spatial ability, creative thinking ability, visual aptitude). Thus, we could easily lead the participants to believe that the test measures an ability that is equally relevant to men and women or one that is less important to men than to women.

The task was presented to the participants either as a test of *visual aptitude* or *language ability*. In the visual aptitude condition, the participants learned that the test is a valid test of visual aptitude and has been shown to predict “visual flexibility, visual potential, and visual accomplishments.” We expected that when the task was described as a visual aptitude test, male and female participants would not differ in the perceived self-relevance of the task to men and women. That is, we expected that male and female participants would perceive the test to be equally self-relevant to men and women. Hence, the perceived self-relevance of the task should be comparable among female and male participants.

In the language ability condition, the participants learned that the test is a valid measure of language ability and predicts “verbal creativity, communication competence, and interpersonal sensitivity.” To suggest to these participants the idea that the “language ability test” measures an ability less valuable to men than women, we emphasized to participants in this condition the importance of language ability to domains stereotypically associated with women: interpersonal relationship, communication with their peers and families, building psychological connections by phone and emails, and becoming a good teacher and school counselor. We expected that in this condition, male participants would perceive the task to be less important to men than to women. Hence male participants would see the task to be less self-relevant.

To ensure that participants would perceive the relevance of the task in the manner we anticipated, we pretested the task and the two instructions (order counter-balanced) to 40 undergraduates (20 females, mean age = 20.07, *SD* = 1.80) who did not participate in the main study. We examined how the two instructions might influence the perceived self-relevance of the test to men and women. Specifically, for each version of the test, participants were asked to indicate (a) how men and women would perceive the importance of the test to the self, (b) how much men and women would care about their test performance, and (c) how much men and women would infer their intellectual ability from their test performance. Participants’ responses were averaged for men and women, separately, to have a composite score of the perceived self-relevance of the test to men and women. The coefficients of the scale were .72 (men) and .75 (women) for the “language ability test” and .83 (men) and .92 (women) for the “visual aptitude test.” As expected, for the “language ability test,” only 1 participant (2.5%) perceived it to be more self-relevant to men than to women; 22.5% of the participants perceived it to be equally important to men and women. The remaining 75% perceived the task to be less self-relevant to men than to women. In contrast, 60% of the participants perceived that the “visual ability test” to be more or equally self-relevant to men (vs. women). In sum, participants in the “language ability test” were successfully manipulated to perceive the task to be more relevant to women than to men, in such a way that they felt that women (vs. men) would be more likely to perceive the task to be important to the self, care about their test performance, and infer their intellectual ability from their test performance. In contrast, participants in the “visual ability test” were induced to perceive the task to be equally relevant to men and women.

### Procedures and Measures

Participants were told that the purpose of the study was to assess their ability in diverse domains. All participants were then given ten minutes to complete the visual pun task without any task description attached to the test. Following Kruger and Dunning [3], to obtain an index of the participants’ actual performance, we assigned each participant to a percentile rank based on their actual performance on the test, relative to the performance of other participants.

After completing the test, the experimenter “debriefed” the participants by presenting some background information on the test they had just completed. At this point, the participants were randomly assigned to the visual aptitude condition or the language ability condition. They were presented with either the visual aptitude task description or the language task description. Next, all participants were asked to estimate their own test performance, compared to the performance of other students at the university, with a percentile rank from 0 (“I’m at the very bottom”) to 100 (“I’m on the top”). As an example, we also explained 50^th^ percentile rank as “I’m better than half and worse than half of other students.” Notice that in this experiment, both the experimental manipulation and the estimation task would have no effect on the participants’ actual task performance. Finally, the participants were fully debriefed and thanked for their participation.

## Results

We hypothesize that perceived task relevance (as manipulated through task framing) would moderate the unskilled and unaware effect. To test our hypothesis, we fitted a 2 (Task Relevance Condition) X Actual Performance (mean-centered) X 2 (Gender: male or female) General Linear Model (GLM) to the participants’ estimated performance on the test. As expected, the predicted three-way interaction was significant, *F*(1, 131) = 5.41, *p* < .05. Age did not affect the results, *Fs*(1, 123) < 1.00, so we dropped that variable in the following analyses. To understand the nature of this 3-way interaction, we fitted an Actual Performance X Gender GLM to estimated performance in the visual aptitude condition and the language ability condition separately.

**Fig 2:**
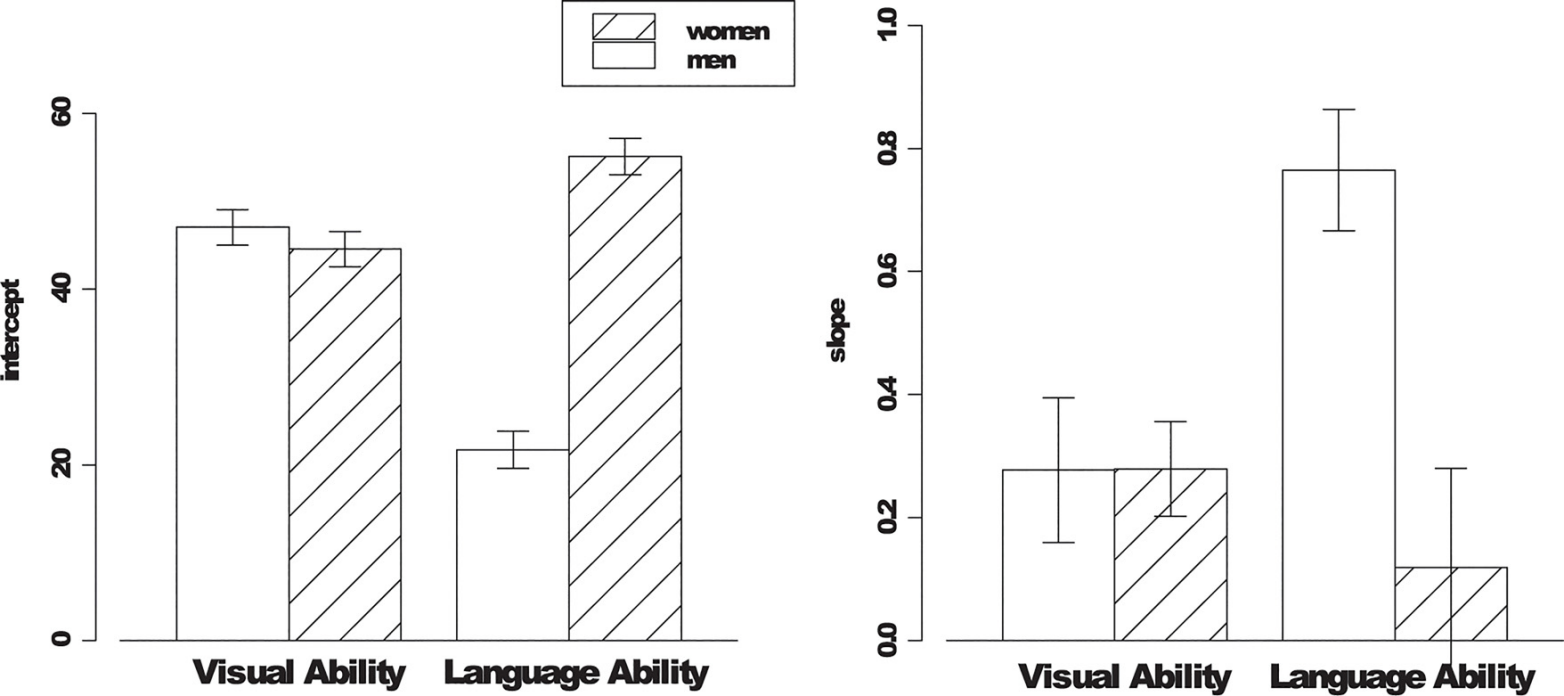
Regression coefficients as a function of gender and task framing.

**Fig 3:**
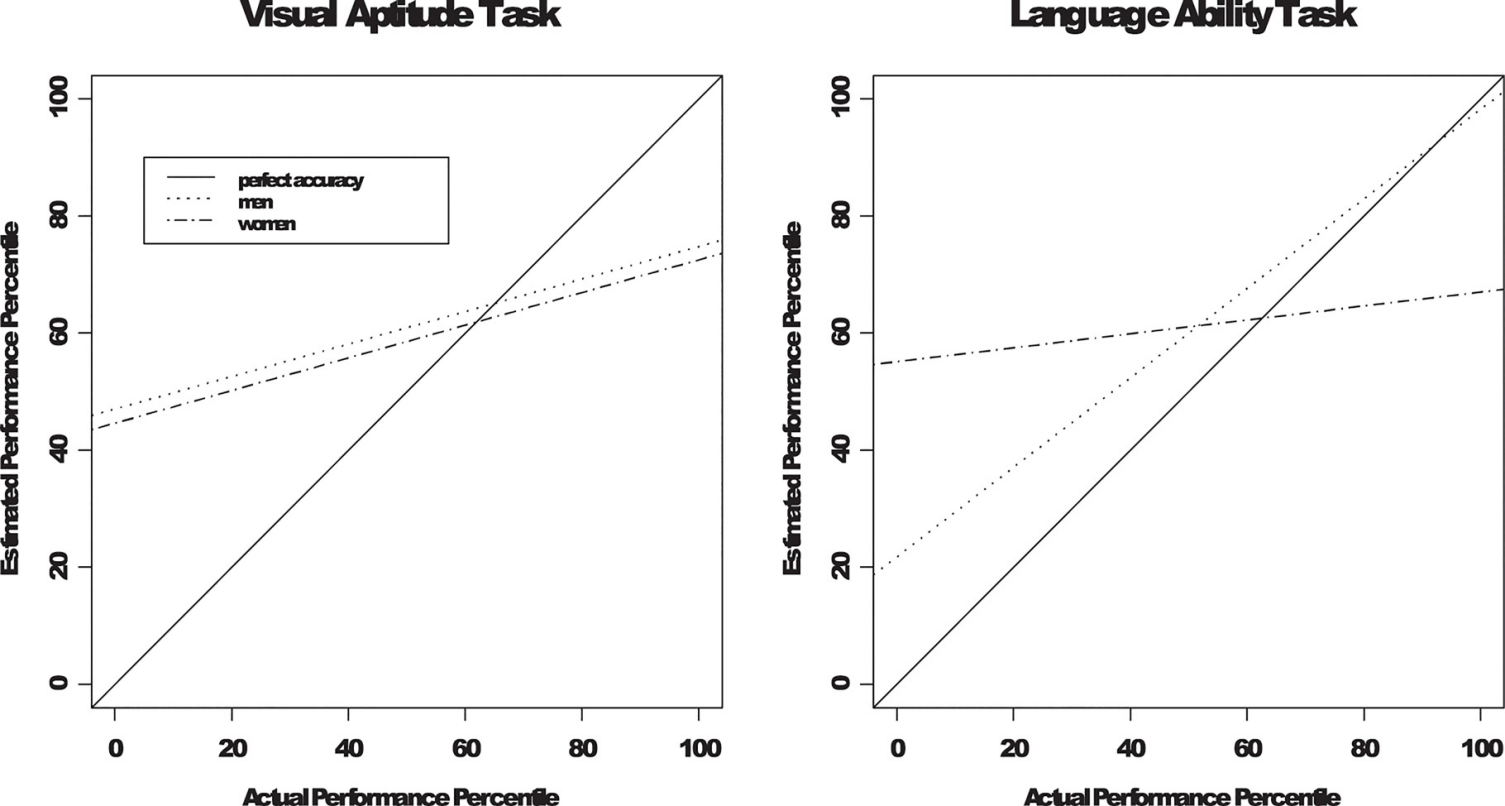
Regression lines in the Gender X Task Framing conditions.

In the visual aptitude condition, the only significant effect on estimated performance was the main effect of actual performance, *F*(1, 92) = 14.52, *p* < .001. Next, we regressed perceived performance on actual performance separately for each gender group in the visual aptitude condition. If the participants had perfect accuracy when estimating their performance, the intercept, which reflects the elevation of the regression line or a positive response bias, should equal zero. Additionally, the slope, which reflects estimation accuracy, should equal one (the unit line). As shown in Fig 2, for both men and women, the intercept was significantly above 0 (47.01 for men and 44.55 for women, *p*s < .05) and the slope was reliably above zero and significantly below 1 (for men, *B* = 0.28, *r* between actual and estimated performance was .37; for women, *B* = 0.28, *r* between actual and estimated performance was .43, *p* < .05). No gender differences in the intercept or the slope were obtained. As shown in Fig 3, replicating the basic results in past research (Kruger & Dunning, 1999; Krueger & Mueller, 2002), participants displayed a positive response bias while at the same time, they had relatively low accuracy when they estimated their performance. Together, these positive response bias and less-then-perfect estimation accuracy jointly contributed to the appearance that low performers overestimated their actual performance and high performers underestimated theirs.

In the language ability condition, the Actual Performance X Gender interaction had a significant effect on estimated performance, *F*(1, 39) = 8.48, *p* < .01. As shown in Fig 2, compared to female participants, male participants had significantly lower intercept (for male, 21.72; for female, 55.10) and a steeper slope (for male, *B* = 0.77, *r* between actual and estimated performance was .88; for female, *B* = 0.12, *r* between actual and estimated performance was .16), indicating a significantly weaker positive response bias and higher sensitivity to their actual performance when estimating their performance.

For men, there was a significant drop in the intercept from 47.01 in the visual aptitude condition to 21.72 in the language ability condition. Furthermore, the Actual Performance X Task Relevance Condition interaction was significant, *F*(1, 52) = 6.24, *p* < .05. The slope in the language ability condition was close to unity (*B* = 0.77, *r* between actual and estimated performance was .88), which was significantly higher than that in the visual aptitude condition (*B* = 0.28, *r* between actual and estimated performance was .37). In short, when the task was perceived to be less relevant to men than to women, men displayed an attenuated positive response bias and tended to base their performance estimation primarily on their actual performance.

Among the female participants, the slope relating estimated performance to actual performance was not significant (*B* = 0.12, *r* between actual and estimated performance was .16, *ns*), *t*(22) = 0.74, *ns*. Furthermore, the Actual Performance X Task Relevance Condition interaction was not reliable, *F*(1, 79) < 1.00, *p* > .05. In short, the results support the hypothesis that perceived self-relevance of the task moderates the unskilled and unaware effect.

## Discussion

Kruger and Dunning (1999) showed that people are not accurate in estimating how their performance compares to others’. Unskilled individuals tend to overestimate their actual percentile of performance and skilled individuals tend underestimate it. We replicated this result in the visual aptitude condition. When the participants were led to believe that the task was equally relevant to men and women, both female and male participants exhibited a significant positive response bias. Their estimated performance was moderately related to their actual performance (*r* = .37 for men and .43 for women). As a result, unskilled performers overestimated their relative performance and skilled performers underestimated it, as illustrated in Fig 3.

Dunning and his colleagues (2003) attributed unskilled performers’ over-estimation of their relative performance to their lack of metacognitive expertise: Unskilled individuals “tend to be blissfully unaware of their incompetence. This lack of awareness arises because poor performers are doubly cursed: Their lack of skill deprives them not only of the ability to produce correct responses, but also of the expertise necessary to surmise that they are not producing them.” (p. 83)

Our results supplement this metacognitive account of the unskilled and unaware effect. If poor performers’ overestimation of their relative performance arises purely from their lack of metacognitive expertise, making them feel that the task is *not* important to the self should not *increase* their estimation accuracy. However, in the language ability condition, when the participants were led to believe that the task was less relevant to male (vs. female) participants, male participants’ positive response bias was significantly attenuated and their estimation accuracy significantly improved, compared to the corresponding results from both male participants in the visual aptitude condition and female participants in the language ability condition. In fact, as shown in Fig 3, the male participants’ estimations of their performance were highly accurate in the language ability condition. It should be emphasized that in the present study, the participants in all conditions performed the same task before the experimental manipulation was introduced. Thus, the results were not due to differences in task nature or the effects of the manipulation on their actual performance.

Similarly, the metacognitive account does not fully explain why female participants exhibited a stronger unskilled and unaware effect than did male participants in the language ability condition. For female participants in this condition, the intercept was 55% and the slope was not significant. Thus, in this condition, most female participants (21 out of 24 females) estimated their performance to be equal to average or above average and these estimations were completely dissociated from actual performance.

The self-enhancement account can explain these results easily. Male participants in the language ability condition did not consider the task to be important to the self. As a result, they were not motivated to self-enhance their performance on the task (see [9]) and therefore did not display a pronounced positive response bias. Instead, they based their estimation primarily on their actual performance. In this case, the unskilled are not motivated to ignore (be unaware of) their incompetence and they estimated their relative performance fairly accurately.

In contrast, female participants in the language ability condition considered the task to be important to the self. Hence, they were motivated to self-enhance; they displayed a pronounced positive response bias and did not base their estimation on their actual performance. In this case, the unskilled are motivated to ignore (be unaware of) their incompetence. As a result, they grossly overestimated their relative performance.

Nonetheless, the study has limitations that could have affected the study findings and interpretations. First, we only manipulated participants to believe that the task was less relevant to men than to women. To have a better understanding of how the perceived self-relevancy of the task impacts people’s estimation of their performance on the task, we should have had another experimental condition where participants were manipulated to believe that the task was less relevant to women than to men. Second, our data is still subject to cognitive explanations. To ensure that female participants in the language ability condition would perceive the task to be relevant to themselves, the pilot test asked participants to indicate how men and women would perceive the relevancy of the task, rather than asking them to indicate how they themselves perceived it. This instruction might have induced participants to perceive the task to be relevant to one’s gender in such a way that female participants inferred that women excel at the task relative to men and they, as women, have performed well on the task. That is, female participants in the language ability condition might have used the task information to infer how well they have performed relative to others.

These results have important implications for predicting psychological well-being and performance. There is considerable evidence that self-enhancement contributes to high self-esteem (e.g., [10]), although the causal link between the two is unclear [11]. Self-enhancement, as part of a system of self-serving illusions, has also been associated with subjective well-being, the ability to care for others, and the ability to be productive or creative [1]. Furthermore, individuals who self-enhance have better physiological reactions to stress–their cardiovascular responses to stressors are less pronounced and they recovered more quickly from stress than those who did not self-enhance [12].

Our results show that when individuals have poor performance in a domain of ability that is important to the self, their self-enhancement motive may drive them to ignore their incompetence. Thus, motivated unawareness of one’s inadequacy in an important ability domain may protect individuals from threats to their self-concept. However, there is also a heavy price for being unskilled and unaware of it. When individuals are unaware of their incompetence, they do not recognize the need for self-improvement. Consequently, they do not see the need to take remedial actions to improve their skills. Hence, their long-term performance may suffer. In support of this idea, we found in our recent research that college students who are unaware of their incompetence in verbal and quantitative abilities have significantly lower GPAs than those who are aware of their incompetence [13]. We also found in another study that college students who are motivated to be unaware of their incompetent experience higher levels of dejection- and agitation-related emotions than those students who are aware of their incompetence [14].

In conclusion, poor performers may possess the metacognitive expertise to recognize their incompetence. However, when the task is perceived to be self-relevant, they are motivated to ignore their incompetence.
